# Estimating the Impact of Reducing Under-Nutrition on the Tuberculosis Epidemic in the Central Eastern States of India: A Dynamic Modeling Study

**DOI:** 10.1371/journal.pone.0128187

**Published:** 2015-06-05

**Authors:** Olivia Oxlade, Chuan-Chin Huang, Megan Murray

**Affiliations:** 1 Department of Epidemiology, Harvard School of Public Health, Boston, Massachusetts, United States of America; 2 Division of Global Health Equity, Brigham and Women’s Hospital, Boston, Massachusetts, United States of America; 3 Infectious Disease Unit, Massachusetts General Hospital, Boston, Massachusetts, United States of America; The University of Tokyo, JAPAN

## Abstract

**Background:**

Tuberculosis (TB) and under-nutrition are widespread in many low and middle-income countries. Momentum to prioritize under-nutrition has been growing at an international level, as demonstrated by the "Scaling Up Nutrition" movement. Low body mass index is an important risk factor for developing TB disease. The objective of this study was to project future trends in TB related outcomes under different scenarios for reducing under-nutrition in the adult population in the Central Eastern states of India.

**Methods:**

A compartmental TB transmission model stratified by body mass index was parameterized using national and regional data from India. We compared TB related mortality and incidence under several scenarios that represented a range of policies and programs designed to reduce the prevalence of under-nutrition, based on the experience and observed trends in similar countries.

**Results:**

The modeled nutrition intervention scenarios brought about reductions in TB incidence and TB related mortality in the Central Eastern Indian states ranging from 43% to 71% and 40% to 68% respectively, relative to the scenario of no nutritional intervention. Modest reductions in under-nutrition averted 4.8 (95% UR 0.5, 17.1) million TB cases and 1.6 (95% UR 0.5, 5.2) million TB related deaths over a period of 20 years of intervention, relative to the scenario of no nutritional intervention. Complete elimination of under-nutrition in the Central Eastern states averted 9.4 (95% UR 1.5, 30.6) million TB cases and 3.2 (95% UR 0.7-, 10.1) million TB related deaths, relative to the scenario of no nutritional intervention.

**Conclusion:**

Our study suggests that intervening on under-nutrition could have a substantial impact on TB incidence and mortality in areas with high prevalence of under-nutrition, even if only small gains in under-nutrition can be achieved. Focusing on under-nutrition may be an effective way to reduce both rates of TB and other diseases associated with under-nutrition.

## Introduction

Tuberculosis (TB) remains a major cause of death and disease in low and middle-income countries (LMIC). The World Health Organization (WHO) attributed 1.3 million deaths and 8.6 million new cases to TB in 2012 [1]. India accounts for the highest burden of TB in the world. In 2012, it had an estimated 2.2 million incident cases [1]. TB cases are not distributed evenly across the Indian population; TB prevalence is over 5 times higher in the poorest wealth quintiles than in the richest [2]. India also suffers from widespread under-nutrition; 17% of the population is undernourished [3] and India is ranked 63^th^ out of 78 countries on the 2013 Global Hunger Index, an indicator that combines undernourishment in the general population, child underweight and child morality [4]. Under-nutrition indicators such as stunting in children are also not equally distributed across the country, and are consistently higher in the more impoverished interior Northern and Central states of India [5].

Previous studies have demonstrated a strong inverse relationship between body mass index (BMI) and TB risk [6]. For example, one recent study that followed health outcomes of a large US population cohort found that those who initially had a BMI of less than 18.5 kg/m^2^ had more than 12 times the hazard of developing TB over the subsequent 20 years compared to those with a normal BMI [7]. Given that 870 million of the 7.1 billion people in the world are chronically under-nourished [3], these data suggest that under-nutrition is a proximal cause of a substantial fraction of the burden of TB in the world [8]. In 2010, the population attributable fraction (PAF) of TB for under-nutrition for the 22 high TB burden countries was estimated to be 27%, higher than the PAF of 16% of HIV for TB [9].

Evidence from many countries suggests that under-nutrition cannot simply be addressed through improved economic productivity [10], and that direct investment in health and health related programs are an important part of the solution in countries like India [11]. In the last decade, a movement to address under-nutrition has gained momentum at both an international and a national level through the implementation and scale up of nutrition specific interventions and nutrition sensitive approaches. Initiatives such as the Scaling Up Nutrition (SUN) [12] movement have galvanized global efforts against under-nutrition [13]. Tackling under-nutrition is now a high priority on the global health agenda [13]. For example, Millennium Development Goal (MDG) 1c is to halve, between 1990 and 2015, the proportion of people who suffer from hunger [14]. Many LMIC are on track to reach this target; the proportion of under-nourished people in developing regions decreased from 23.2% to 14.9% between 1990 and 2012 [15]. The impact of improving nutritional status among the world’s poor is likely to be far-reaching and one expected effect will be a reduction in TB incidence and TB related mortality. The objective of this study was to predict future trends in TB related outcomes under different scenarios for reducing under-nutrition and increasing BMI, in the general adult population in the poorest states of India.

## Methods

### Ethics Statement

An ethics exemption waiver was issued by Harvard School of Public Health as the Demographic Health Survey (DHS) data set is a public data set and all other data used in models is previously published.

### Setting

**Fig 1:**
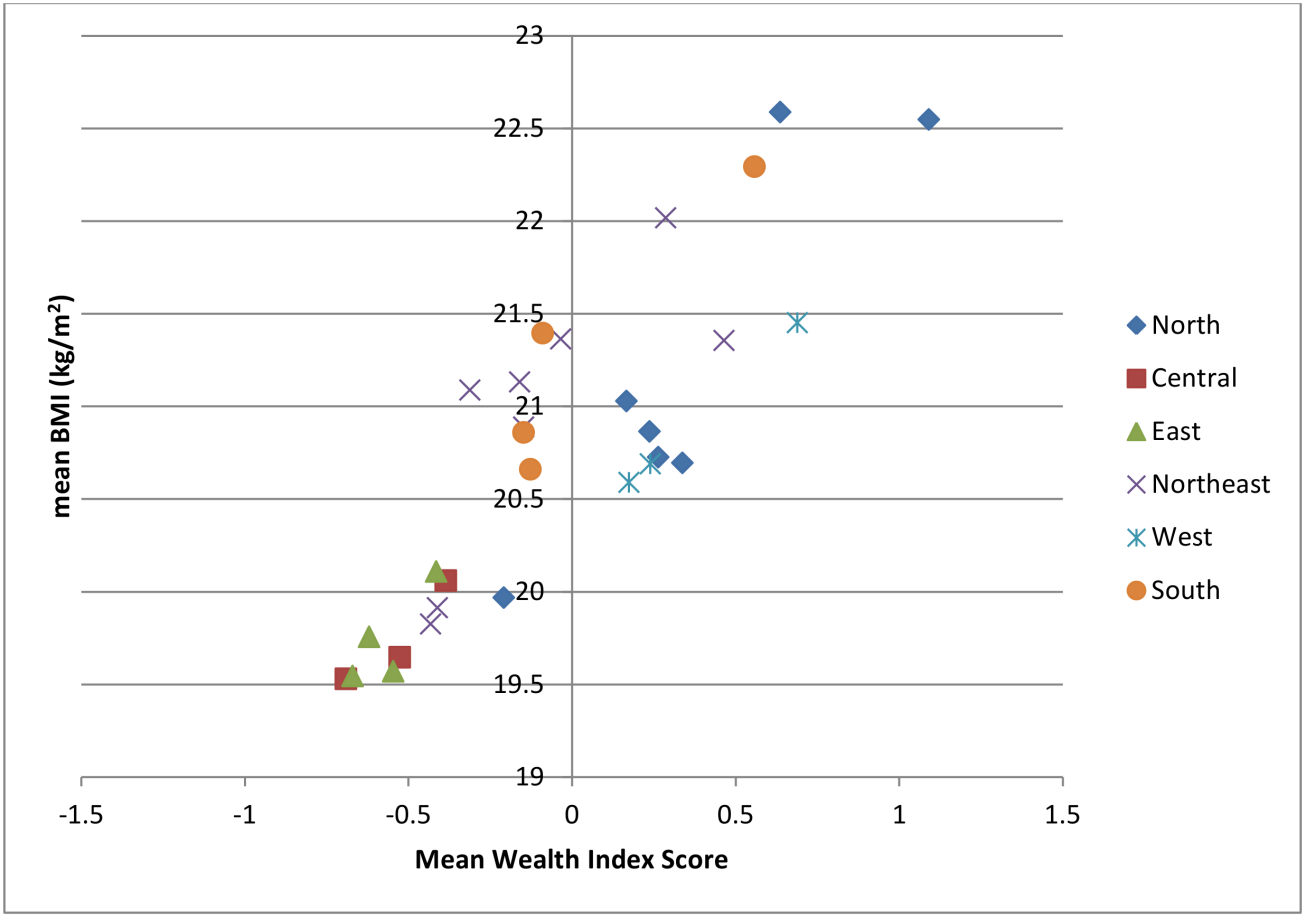
Association between mean BMI (kg/m^2)^ and mean Wealth Index Score, by Demographic Health Survey (DHS) survey region in India.

We chose to model nutritional changes in the Central Eastern States of India as a case study because of the high rates of both TB and under-nutrition in these regions [16] and the relatively low rate of HIV infection [17]. The Central, East and North Eastern States of India (including the following States: Arunachal Pradesh, Bihar, Meghalaya, Assam, West Bengal, Nagaland, Jharkhand, Manipur, Uttar Pradesh, Mizoram, Madhya Pradesh, Sikkim, Orissa, Tripura and Chhattisgarh) were amongst the poorest Demographic Health Survey (DHS) defined geographical regions in India in the period 2005–6 [16] and had amongst the lowest mean BMIs (Fig 1). The mean BMI, 20.58 kg/m^2^ [16], in this region is comparable to the mean BMIs of other LMIC with a high prevalence of under-nutrition such as Bangladesh and Ethiopia (mean BMIs: 20.32 kg/m^2^ and 20.48 kg/m^2^ respectively [18]).

### Dynamic TB model

We used a deterministic dynamic SLIR (“Susceptible-Latent-Infectious-Recovered”) model similar to those described elsewhere [19] to predict the impact of reducing under-nutrition on future trends of TB (See S1 File and Fig A in S1 File). We based model parameters on previous epidemiological studies on the natural history of tuberculosis and low BMI, and on demographic parameters specific to India. The transmission parameter was calibrated to trends in reported TB incidence in India which were derived from State specific TB prevalence data in 2005 [16], and time trends from national data, as reported by the WHO [20]. We introduced under-nutrition into the model by stratifying the model population into four exposure levels defined by the mean BMI for each quartile as reported in 2005–6 DHS [16] (See Fig B in S1 File). We subsequently allowed the proportion of the population in each stratum to vary over time in order to represent different scenarios of reduced under-nutrition.

### Data sources and Model calibration

We first fit a fixed transmission parameter calibrated to the equilibrium TB incidence reported by the WHO for the years 1990 to 2000 and then allowed this parameter to decline linearly between the years 2000 and 2010 to correspond to the reported decline in TB incidence during this period. We assumed that incidence will continue to decline at this rate until 2030.

The effective reproduction number is a function not only of the transmissibility of the organism but also of the average time period during which TB patients are infectious. This period reflects both case detection rates and treatment outcomes including death and treatment failure. Data on the rates of TB-specific mortality and treatment failure in the public sector came from the 2013 WHO Global TB Report. However, because almost 50% of TB cases in India are reported to be detected and treated within the private sector [21], we used published reports on TB outcomes in the private sector to calculate weighted averages for the case detection rate and the proportion of cases completing treatment [20] [22]. We based our estimates of the total population for the region on the 2011 India census [23] and modeled projected population growth between 2010 and 2030 using estimates from the United Nations [24]. All model parameters are shown in Tables 1 and 2. We applied the calibrated model to estimate future TB incidence and mortality under different scenarios of reduced under-nutrition.

**Table 1:** Time varying model transitions- Value at start of period of decline (2000).

Parameter Description	Value in 2000	Range (+/- 10% of point estimate)	Reference
Per capita transmission parameter	3.93 E-06	-	Fitted to TB incidence data during period of equilibrium
linear trend in transmission parameter (between 2000–2030)	-5.91E-08	-6.5E-08, -5.3E-08	Fitted to TB incidence data during period of improvement (2000–2011)
Probability of treatment success for those starting TB treatment	0.771	-	Weighted average of public and private sector. [22] [20] [21]
linear trend in probability of treatment success (between 2000–2030)	0.0032	0.00288,0.00352	Derived from WHO data between 2000–2011 [20]
Probability of failure/default for those starting TB treatment	0.189	-	Calculated from probability of TB treatment success and mortality
trend in probability of failure/default (between 2000–2030)	Dependant on change in probability of treatment success and death	-	Calculated from probability of TB treatment success and mortality
Birth rate	0.0267/yr	-	[24]
linear trend in birth rate (between 2000–2030)	-0.0004	-0.00044, -0.00036	[24]
Population Growth Rate	0.0104/yr	-	[24]
linear trend in Growth rate (between 2000–2030)	-0.0003	-0.00033, -0.00027	[24]
Background mortality rate	0.0163/yr	-	Calculated from Population Growth rate and Birth rate
trend in mortality rate (between 2000–2030)	Dependant on change in population growth and birth rate	-	

**Table 2:** Time invariant model transitions.

Parameter Description	Value	Range*	Reference for baseline
Primary progression rate for fourth quartile(see Table 3 for rates by BMI strata)	0.03/yr	0.0225–0.0375	[50] [51]
Reactivation rate for fourth BMI strata(see Table 3 for rates by BMI strata)	5.0E-05/yr	4.5 E-05, 5.5 E-05	[51]
Transition rate from primary to reactivation	0.2/yr	0.15–0.25	[50]
Death rate untreated TB	0.3/yr	0.225–0.375	[51] [52]
Natural cure rate	0.2/yr	0.15–0.25	[53]
Partial immunity that decreases probability of fast progression after re-infection	0.5	0.3–0.5	[50] [53]
TB Diagnosis rate	0.389/yr	0.35, 0.43	[20] [21] [54]
Probability of death for those starting TB treatment	0.038	0.0342, 0.0418	Weighted average of public and private sector. [22] [20,21]

*Range taken from estimates presented in the literature or +/- 10% of point estimate if no additional published estimates available

### Data sources for risk-factor effect sizes

Current data suggests that low BMI increases the risk of reactivation and progression to active disease from latent infection but does not alter susceptibility to TB infection among those exposed. Lonnroth et al. reported a consistent inverse log linear relationship between BMI and TB incidence for BMIs that ranged between 18.5 kg/m^2^ and 30 kg/m^2^ [6]. We used data from the six studies reviewed in Lonnroth et al. to create a prediction model for the association between TB incidence and BMI after replacing the data from one unpublished data set with a more recent published version of the data [7]. We restricted the analysis to data points for BMIs < = 27 kg/m^2^ because the mean BMI in the highest quartile in our population was only 25 kg/m^2^. We fit and compared two separate models for this association. First, we regressed log-transformed TB incidence on BMI and included an indicator variable to account for different TB incidence values at baseline BMI in studies from different populations. Second, we used a penalized spline for BMI in the regression, choosing the smoothing parameter using the generalized cross validation criterion [25]. The penalized spline model was chosen to better capture the association in non-linear data, although results were very similar between the log transformed and spline models. Using the spline model we predicted the TB incidence rate for the mean BMI for each quartile in 2005 in our study population (BMI range 16.59 kg/m^2^ to 25.06 kg/m^2^ [16]), and then estimated the following relative risks for BMI quartiles one to three- compared to the fourth quartile: 4.95, 3.00 and 2.25 (Table 3). The 95% confidence intervals of risk estimates were calculated using a bootstrap method.

**Table 3:** Estimates of impact of Body Mass Index on rates of reactivation of long standing TB infection and rapid progression to TB disease.

BMI Strata*	Estimate of effect and 95% Confidence Interval	Reactivation rate	Progression rate
FIRST (< = 17.8)	4.95 (3.54–6.56)	0.025%	14.85%
SECOND (17.8 to < = 19.64)	3.00 (2.73–3.83)	0.015%	9.00%
THIRD(19.64 to < = 22.25)	2.25 (1.88–2.68)	0.011%	6.75%
FOURTH (> 22.25)	REFERENCE	0.005%	3.00%

*BMI quartiles at start year. Calculated from DHS 2005 data from Central Eastern Indian States

### Scenarios of reduced under-nutrition

Given the complex social determinants involved in reducing under-nutrition, stakeholders suggest that multi-sectoral approaches supported by high-level political commitment will be required to reduce under-nutrition. The Scaling Up Nutrition (SUN) movement supports country led action against under-nutrition and is governed by a group of heads of state and other key stakeholders. Initiated in 2010, SUN now includes 54 countries in which national leaders have committed to prioritizing multi-sectoral efforts to address under-nutrition [12]; in 2013, 15 of these countries demonstrated an average annual rate of reduction in stunting prevalence of more than 2% per year [26].

Although only one Indian state (Maharashtra) is currently enrolled in the SUN movement, several recent developments suggest that the progress observed in other countries could be replicated in India; the Government of India doubled public spending on health between 2004 and 2009 [27]. They have continued to support the Indian Mid-day Meals program in schools, initially implemented in 2002 and now reaching 120 million children nationally, it is considered to be one of the most wide reaching and successful interventions by the Indian Government in recent years. The program was recently evaluated and shown to have led to significant gains for families that have suffered have from drought [28]. Most recently, in 2013, India passed a National Food Security bill that legislates the provision of subsidized food grains to approximately two thirds of India's 1.2 billion people [29,30]. This bill also designates women as heads of household empowered to hold ration cards. Other national multi-sectoral initiatives recently undertaken in India include the “Multi-Sectoral Nutrition Programme to Address Maternal and Child Under-nutrition” [31] which will implement core nutritional interventions in 200 high burden districts. More information on the most important nutrition sensitive and specific factors in India is provided in the technical appendix (S1 File).

For our nutritional scenarios, we constructed several possible scenarios that represent a range of policies and programs designed to reduce the prevalence of under-nutrition based on the recent experience and observed trends in different countries (Table 4 and S1 File). Scenarios were selected based on achievements that have been made in three case study countries (Bangladesh, Ghana and Vietnam) that are part of the SUN movement. These three countries are amongst those that have demonstrated government commitment to tackling under-nutrition at a country level, have developed successful nutrition sensitive and/or specific programs and have shown a sustained improvement in both nutritional related as well as more general development related indicators.

**Table 4:** Summary of Evidence for countries selected as case studies to inform nutritional interventions scenarios.

	Bangladesh	Vietnam	Ghana
*Progress towards MDG 1c [4,14])* (To halve, between 1990 and 2015, the proportion of people who suffer from hunger)			
Indicator used to measure MDG: Proportion of population falling below minimum level of dietary energy consumption. % decline in indicator per year between 1990–2010 [4]*	4.6%	8.0%	11.7%
*Determinants of Under-nutrition [36]* (absolute change (%) per year between approximately 1990–2010)			
Health expenditure per capita (current US$)	0.87	3.68	5.20
Literacy rate, adult female (% of females ages 15 and above)	1.38	1.55	0.37
Poverty gap at $1.25 a day (PPP) (%)	0.25	0.33	-0.00733
Employment in agriculture (% of total employment)	-0.70	-0.60	-1.32
Under-nutrition related policies, programs and actions between 1990 and 2010: [55]			
*Examples of relevant country level commitment and policies*	-National Food and Nutrition policy (1997)	-National Plan of Action for Nutrition (1995)	-National Plan of Action on Food and Nutrition (1995)
	-Bangladesh National Plan of Action for Nutrition (1997)	-National Nutrition Strategy (2001)	-Imagine Ghana Free of Malnutrition (2005)
	-National Food Policy (2006)	-National Nutrition Action Plan (2006)	-Growth and Poverty Reduction Strategy (2006)
	-National Health Policy (2008)		
	-National Food Policy Plan of Action (2008)		
	-National Agricultural Policy (2010)		
*Examples of relevant country level programs and action*	-Maternal, infant and young child nutrition programs. Food distribution/supplementation (lactating women, pregnant women) (date unknown)	-The Protein-Energy Malnutrition (PEM) Control Program Complementary feeding promotion and/or counseling (Infants and young children) (1994)	-Maternal, infant and young child nutrition programs. Food distribution/supplementation (Infants and young children) (2010)
	-Maternal, infant and young child nutrition programs. Prevention or treatment of moderate malnutrition (preschool-age children) (date unknown)	-The Protein-Energy Malnutrition (PEM) Control Program. Food distribution/supplementation (1994)	-Assistance to Ghanaian Food-Insecure Households in Northern Ghana Food distribution/supplementation (HIV cases, infants and young children, lactating women & pregnant women) (2010)
			-Purchase for Progress (P4P) Pilot Initiative, Conditional cash transfer (Adult men and women) (2008)
			-Food Security and Environment Facility Promotion of food security and agriculture (Adult men and women) (2008)

* Under-nutrition related indicator used to Inform rate of change in BMI in each nutritional intervention scenario (See Table 5)

In scenario 1, we assume no improvement in the current prevalence of under-nutrition consistent with the pattern of under-nutrition in adults in India observed between the DHS in 1998 and 2005 [16], the observed decline in BMI in men in during this period [32] [33], as well as the lack of change in stunting and wasting in children in India between 1992 and 2005 [16]. In scenario 2, we assume reductions based on the experience of Bangladesh, which began by strengthening its political commitment to combat under-nutrition with the implementation of policies such as the National Food and Nutrition Policy in 1997 and implementing country level nutrition programs such as the prevention and treatment of moderate malnutrition program in pre-school age children [12]. In scenario 3, we assume deeper reductions consistent with those observed in Vietnam where a National Plan of Action for Nutrition was first introduced in 1995. In Vietnam, specific programs that have been implemented include complementary feeding promotion and nutritional counseling for mothers of infants and young children [12]. In scenario 4, we assume reductions consistent with the best empirical case based on the experience of Ghana which has a long history of government commitment to under-nutrition, including initiatives such as the National Plan of Action on Food and Nutrition (1995–2000). Many wide reaching nutrition programs have been implemented in Ghana including food distribution to food insecure households, conditional cash transfer programs, as well as programs that focus on the promotion of food security and agriculture [12]. This scenario is considered an under-nutrition elimination scenario as less than 5% of the population remained in the lowest BMI strata after 20 years; and finally in scenario 5, we model improvements to under-nutrition as we have described in scenario 2 but also consider further improvements to the TB treatment program beyond those predicted by current trends (up to 97% treatment success by 2030 versus 87% in other scenarios).

### Model Modifications for Scenarios of Reduced Under-nutrition

Limited data are available on expected changes in adult BMI in response to nutritional interventions. To estimate future trends in BMI under each nutritional intervention scenario, we used an indicator of improving nutrition from each of the case study countries- the change in the proportion of the population classified as malnourished (falling below minimum level of dietary energy consumption) as reported by the Food and Agriculture Organization of the United Nations (FAO) [4]. For each nutritional intervention scenario (summarized in Table 5), we allowed the modeled population to transition between BMI categories based on the annual rates of change in the prevalence of under-nourishment reported by the FAO between 1990 and 2010 (as shown in Table 4). The proportion of the population in the higher BMI strata thus increases over time, however mean BMI values for each strata do not change and are based on 2005 DHS data.

**Table 5:** Summary of scenarios for nutritional interventions.

Scenario 1: No change in the prevalence of population undernourished (minimal improvements to TB program)
Scenario 2: Achieve average annual change in prevalence undernourished equivalent to Bangladesh (4.6% per year increase across BMI strata), from 2011–2030
Scenario 3: Achieve average annual change in prevalence undernourished equivalent to Vietnam (8.0% per year increase across BMI strata), from 2011–2030
Scenario 4: Achieve average annual change in prevalence undernourished equivalent to Ghana (11.7% per year increase across BMI strata), from 2011–2030
Scenario 5: Achieve average annual change in prevalence undernourished equivalent to Bangladesh plus double rate of improvement of TB treatment success, from 2011–2030

### Sensitivity Analysis

We conducted sensitivity analysis by sampling from a uniform distribution for each parameter. Where possible, ranges of values from the literature or 95% confidence intervals around parameters were used to define distributions. If this information was not available from the literature a range of +/- 10% around the point estimate was used. 95% uncertainty ranges are reported as the 2.5^th^ and 97.5^th^ percentiles of results from 1000 trials.

## Results

**Fig 2:**
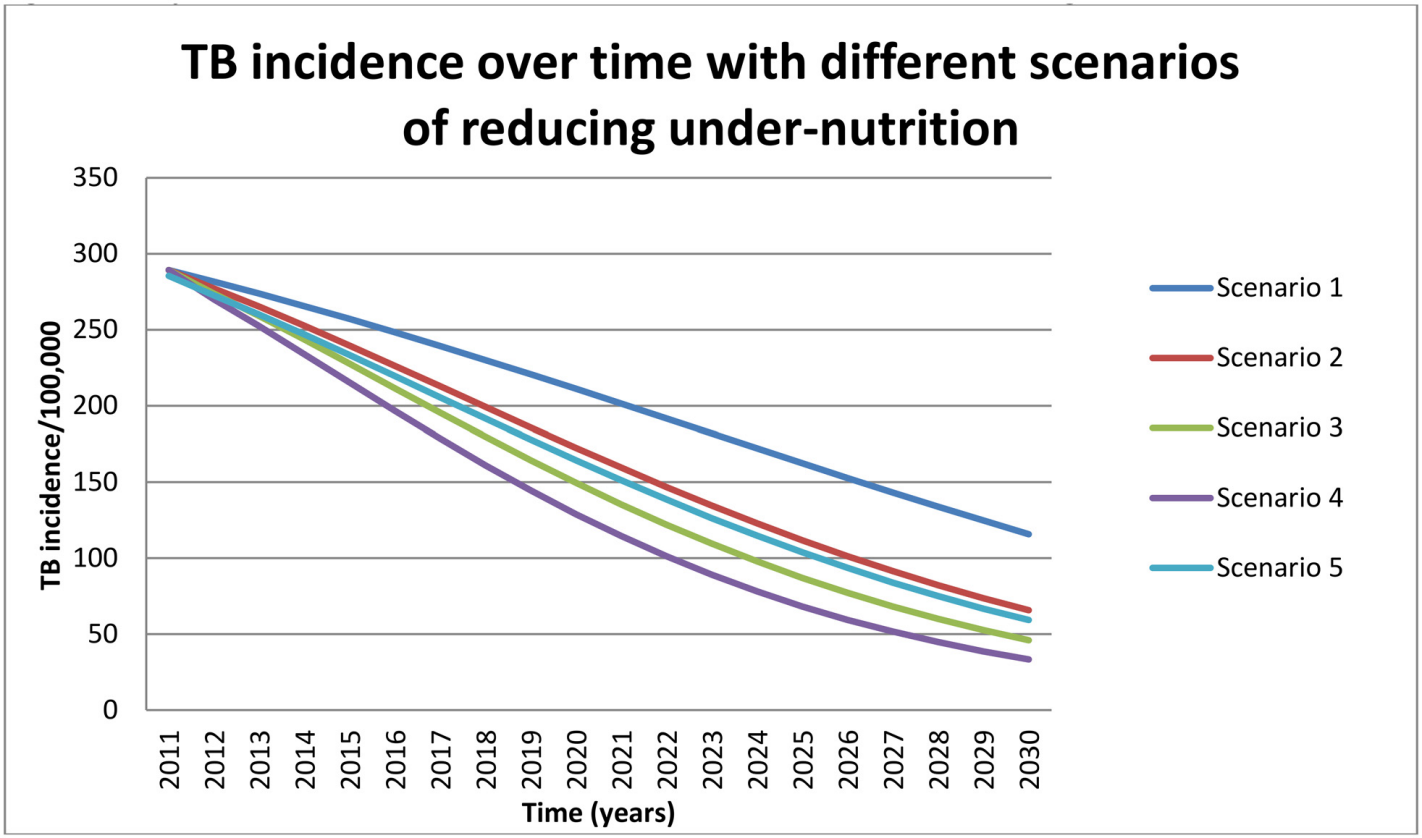
Projected TB incidence over time with different scenarios of reducing under-nutrition.

If the current level of TB case detection is maintained and outcomes continue to improve at the current pace, TB incidence and mortality in the Central Eastern States of India is expected to decline (Fig 2) over20 years from 289 (95% UR 108, 590) per 100,000 in 2011 to 116 (95% UR 20, 464) per 100,000 in 2030 and from 110 (95% UR 45, 207) per 100,000 to 46 (95% UR 8, 54) per 100,000 respectively, even in the absence of any observed reduction in under-nutrition. This translates to expected TB incidence and TB related mortality rates in the lowest BMI strata of 177 (95% UR 28, 694) per 100,000 and 71 (95% UR 12, 276) per 100,000 respectively in 2030, compared to 54 (95% UR 9, 210) per 100,000 and 21 (95% UR 4, 77) in the highest BMI strata.

Table 6 shows estimated reductions in the TB incidence rate by 2030 under the different scenarios of improved nutritional status; these range from 43% (reduced to 66 (95% UR 14, 275) per 100,000) for more modest reductions in under-nutrition (scenario 2) to 60% (reduced to 46 (95% UR 8, 188) per 100,000 in scenario 3 and 71% (reduced to 33 (95% UR 7, 140) per 100,000) in the scenario where under-nutrition is eliminated (scenario 4). Reductions to the TB related death rate (Table 7) are expected to be of similar magnitude; 40% (reduced to 27 (95% UR 5, 96) per 100,000) with scenario 2, and as high as 68% (reduced to 15 (95% UR 3, 53) per 100,000) for the elimination scenario (scenario 4). Tables 8 and 9 show the absolute number of TB cases and deaths averted in the Central Eastern states in 2030 under each of the scenarios.

**Table 6:** TB Incidence rate per 100,000 in Central Eastern India in 2030, with different nutritional scenarios in place between 2010–2030.

Nutritional Intervention	TB incidence rate per 100,000 (95% UR)	% Reduction versus No Intervention
Scenario 1	116 (20,464)	-
Scenario 2	66 (14,275)	43%
Scenario 3	46 (8,188)	60%
Scenario 4	33 (7,140)	71%
Scenario 5	59 (10,232)	49%

**Table 7:** TB Mortality rate per 100,000 in Central Eastern India in 2030, with different nutritional scenarios in place between 2010–2030.

Nutritional Intervention	TB mortality rate per 100,000(95% UR)	% Reduction versus No Intervention
Scenario 1	46 (8,172)	-
Scenario 2	27 (5,96)	40%
Scenario 3	20 (4,73)	57%
Scenario 4	15 (3,53)	68%
Scenario 5	24 (5,88)	48%

**Table 8:** Number of TB cases and TB cases averted (versus no intervention) in Central Eastern India using TB incidence rate in 2030, with different nutritional scenarios in place between 2010–2030.

Nutritional Intervention	Number of TB Cases in 2030 by BMI Strata				Full population (95% UR)	Cases Averted (versus no intervention) (95% UR)
	First	Second	Third	Fourth		
Scenario 1	310,570	229,392	187,551	98,053	825,566 (131,058–3,274,762)	-
Scenario 2	87,609	124,113	124,681	131,658	468,061(85,598–1,823,535)	357,504(45,460–1,451,227)
Scenario 3	34,442	67,880	84,650	140,816	327,788(61,218–1,322,204)	497,777(69,839–1,952,558)
Scenario 4	12,495	32,792	51,263	141,438	237,988(48,599–947,438)	587,578(82,459–2,327,324)
Scenario 5	78,602	111,758	112,550	119,459	422,369(79,948–1,693,991)	403,196(51,110–1,580,771)

**Table 9:** Number of TB deaths and TB deaths averted (versus no Intervention) in Central Eastern India using TB mortality rate in 2030, with different nutritional scenarios in place between 2010–2030.

Nutritional Intervention	Number of TB deaths in 2030 by BMI Strata				Full population (95% UR)	Deaths averted (versus no intervention) (95% UR)
	First	Second	Third	Fourth		
Scenario 1	123,728	91,111	74,375	38,761	327,975 (58,467–1,198,470)	-
Scenario 2	38,413	52,655	51,896	52,608	195,571(34,926–722,184)	132,403 (23,542–476,286)
Scenario 3	16,135	30,480	36,816	57,190	140,622 (27,595–533,615)	187,353 (30,873–664,855)
Scenario 4	6,261	15,660	23,486	58,484	103,891(20,696–380,539)	224,084 (37,772–817,930)
Scenario 5	32,941	45,371	44,868	45,764	168,944(31,762–638,701)	159,031 (26,706–559,769)

Over the 20 years of the modeled intervention, we estimate that 4.8 (95% UR 0.6, 17.1,) million cases and 1.6 (95% UR 0.5, 5.2) million deaths could be averted with scenario 2 while 7.3 (95% UR 1.2, 22.2) million cases and 2.9 (95% UR 0.5, 7.1) million deaths could be averted in scenario 3 and, 9.4 (95% UR 1.5, 30.6) million cases and 3.2 (95% UR 0.7, 10.1) million deaths could be averted with scenario 4 (Table 10).

**Table 10:** Cumulative number of TB cases and TB deaths averted in Central Eastern India with different scenarios of reducing under-nutrition (versus no intervention) over 20 years.

Nutritional Intervention	Total Cases (95% UR)	Cases Averted(95% UR)	Total Deaths (95% UR)	Deaths Averted(95% UR)
Scenario 1	28,330,624 (6,804,752–81,277,665)	-	10,947,552 (2,997,107–29,354,217)	-
Scenario 2	23,503,738 (6,233,031–64,205,503)	4,826,886 (571,721–17,072,162)	9,349,558 (2,534,781–24,152,436)	1,597,994 (462,326–5,201,781)
Scenario 3	20,975,553 (5,628,148–59,037,939)	7,355,070 (1,176,604–22,239,726)	8,491,363 (2,520,961–22,209,764)	2,456,189 (476,146–7,144,453)
Scenario 4	18,882,052 (5,343,598–50,653,542)	9,448,571 (1,461,154–30,624,123)	7,768,496 (2,322,116–19,268,098)	3,179,056 (674,991–10,086,119)
Scenario 5	22,530,504 (6,101,427–63,381,267)	5,800,120 (703,325–17,896,398)	8,759,050 (2,479,705–23,250,291)	2,188,502 (517,402–6,103,925)

We also considered the impact of these interventions in the setting of concurrent improvements to the TB program (scenario 5) (Tables 6 and 7). In this scenario, TB incidence in 2030 improved marginally relative to scenario 2, to 59 (95% UR 10, 232) per 100,000. Similarly, TB mortality in 2030 only decreased by a small amount relative to reductions achieved in scenario 2. As shown in Fig 2, attempts to reduce TB incidence through simultaneous improvements in both under-nutrition and TB treatment programs are predicted to achieve very little relative to the impact that can be achieved by focusing exclusively on under-nutrition.

## Discussion

These results suggest that intervening on under-nutrition could have a substantial impact on TB mortality and incidence in LMIC with a high burden of under-nutrition. Even if interventions lead to relatively small reductions in under-nutrition, close to 5 million cases and 1.6 million deaths could be averted in the Central Eastern States of India over a twenty-year period of sustained improvements in nutrition. This finding adds to the growing literature that demonstrates that substantial gains could be achieved toward reducing TB rates by intervening on risk factors that also underlie other conditions and that increase early mortality [34] [9]. A recent study from Odone et al. [35] reported that complete eradication of under-nutrition globally would lead to a further decline in TB incidence of 18% by 2035 relative to their base case. These projections are more conservative than what we report, possibly because this study focused on global changes rather than on a specific sub-population with a very high risk of under-nutrition.

In India, where high rates of under-nutrition persist among the poor, interventions directed at under-nutrition could have a substantial effect on the TB epidemic. However, the question of how nutritional programs will be shaped and funded in India remains. In the past several decades improvements to nutrition related determinants have been slow, and political commitment to targeted programs have been inconsistent. However, recent evidence suggests that a more substantial political commitment is being made to under-nutrition. For any gains in under-nutrition to be realized, an appropriate country specific approach or program would have to be carefully developed and implemented, requiring both sustained political support and dedicated funds [36]. For interventions to have a substantial and far reaching impact, evaluation of feasibility and scalability of relevant interventions across different regions of India would be required. In addition, the cost effectiveness of implementing nutritional interventions, which was not included in our analysis, would need to be carefully considered as part of the program selection process in India.

Targeting under-nutrition and adult BMI could be expected to have an impact on TB cases and deaths in many other settings; of the 22 high TB burden countries with data on rates of under-nutrition, almost 60% are included in the 50 countries with the highest burdens of undernourishment [4]. For example, Ethiopia, Mozambique and Tanzania all rank within the 10 countries with the highest prevalence of under-nutrition, with proportions of under-nutrition of 40.2%, 39.4%, and 38.8% respectively and TB prevalence rates in 2012 of 224, 553 and 175 per 100,000 respectively [1,4].

The declines in TB incidence estimated in this study parallel the experience of many Western countries during the last century [37,38]. For example, TB incidence and mortality fell by 5.4% per year in the Netherlands between 1910 and 1940 [37,38] and these reductions occurred prior to the introduction of treatment or the wide-spread use of BCG vaccine. McKeown and others have proposed that social and economic changes that led to reduced overcrowding and improved nutritional status were responsible for the rapid decline in TB outcomes in the absence of effective intervention [38] [37,39] [40]. The 2%-5% per year reduction in TB incidence between 2010 and 2030 (calculated from data shown in Fig 2) that we have estimated in the poorest regions of India is consistent with the hypothesis that much of the decline in TB incidence experienced in countries like the Netherlands and the United Kingdom during the pre-chemotherapy era may have been attributed to improvements in nutrition.

Our study has several limitations. First, we modeled under-nutrition in isolation of other social determinants and risk factors in order to understand how the impact of a nutritional program might reduce TB incidence and deaths. Although low BMI is often associated with other risk factors for TB such as biomass fuel use [41], and micronutrient deficiencies [42], we believe the risk ratios for the association between BMI and TB derived from published data [6] mostly reflect the impact of BMI rather than the other risk factors with which low BMI is often correlated. Studies included in the systematic review where data was derived to obtain risk estimates used in our study had all controlled for various sets of confounders, and the relationship observed was remarkably consistent across studies, including those that controlled for confounders such as smoking, diabetes, alcohol, crowded living conditions etc. In addition, several of the studies included in the review were carried out in high income countries where confounders such as biomass fuel exposure would be less common [6]. Nevertheless,, we cannot be certain that the impact of these risk factors would not have influenced our projections and reduced the impact of the nutritional inventions we considered in our model. For example, if improving BMI leads to general improved health and increased productivity, other poverty-related risk factors may also be reduced, thereby further lowering TB risk. A thorough understanding of how BMI interacts with many of these associated risk factors would be essential when considering the design of specific interventions aimed at increasing BMI in this setting.

Second, due to the lack of data that links nutritional interventions with increased BMI in adults, we assumed that rates of change in BMI after interventions follow temporal trends from 1990 to 2010 in the proportion of the population that was undernourished as reported by the FAO for the case study countries [4]. Some observational data from small studies suggests that malnourished individuals require approximately 5,000 to 10,000 excess Kcal to gain one kilogram of weight [43]. However, the actual rate at which individuals gain weight over time likely depends on both their initial BMI, body composition and the duration of time over which they have been receiving additional calories [43,44]. In the absence of more detailed and generalizable data, we made the following simplifying assumptions. First, we assumed that the rate of change in adult BMI during an intervention remains constant over time, and second, we assumed a homogeneous effect across the population, that is not dependent on initial BMI. These assumptions could mean that our estimates of the impact of nutrition related interventions have been over estimated, although without further information on both our study population, and the type of intervention that we are assuming, the specific effect is difficult to predict. We also may have exaggerated the impact of nutrition related interventions if poor nutrition in childhood has lifelong effects that cannot be remedied with further nutritional supplementation later in life. However, although evidence suggests that children who are stunted tend to grow up to be stunted as adults, a window exists during adolescence when some height deficit can be made up [45]. Beyond this period, the height of adults is not likely to change, but it is most likely that additional food will increase the weight of adults, which will in turn increase BMI, as we have assumed in our scenarios.

Third, we assumed that mixing in the population in all four BMI strata occurred at random. In reality, assortative mixing of individuals is more likely, which would likely further amplify the impact of scenarios considered. Fourth, data on trends in TB incidence over time were only available at a national level. If trends differ at a sub-national level, then some bias could result in our projections. Finally, there is ongoing uncertainty about the future role of the private sector in TB control in India. We have therefore assumed that the treatment success rate improved gradually over time, but that the case detection rate remained constant for the duration of the interventions. Although it is possible that case detection will improve in the public sector, the private sector continues to be heavily used in India and minimal improvements to physician practices in the private sector have been noted in recent years [46,47] [48], meaning that our estimates for improved treatment success used in all scenarios may be overly optimistic.

Other modeling studies have considered nutritional changes in India [33,49] and globally [35] and have highlighted the dual burden of both under-nutrition and Diabetes Mellitus (DM) on TB risk, suggesting that DM is an important driver of the burden of TB in the general population. In our study, we did not extend our model to include DM because our analysis was restricted to the poorest states in India, with mean BMIs of 20.6 kg/m^2^ and thus, even with interventions aimed at increasing BMI, this population is at low risk of developing DM later in life. Furthermore those with high BMI who do not develop DM are at substantially reduced risk for TB, thereby mitigating any effect of increasing DM risk [6].

There is now ample evidence from SUN countries that long-term political commitment as well as direct investment in targeted health and health related programs can lead to sustained change through many different nutrition specific and or sensitive programs. As global momentum related to under-nutrition continues to increase it remains to be seen if a sustained commitment can be made to combat under-nutrition in India. We predict that in India, nutrition could be a very worthwhile area for investment- both from the perspective of TB control, as well as the many other diseases affecting populations living in poverty, that are affected by nutritional status and the availability of food.

## Supporting Information

S1 File Supporting figures. Fig A in S1 File. Compartments and rates for TB transmission model. Fig B in S1 File. TB transmission model stratified by Body Mass Index.
(DOCX)
