# Supplementary material for: Estimating the Impact of Reducing Under-Nutrition on the Tuberculosis Epidemic in the Central Eastern States of India: A Dynamic Modeling Study
Source: PLoS One. 2015 Jun 5;10(6):e0128187. doi: 10.1371/journal.pone.0128187 (PMC4457886; doi:10.1371/journal.pone.0128187)
Supplement: S1 File — Fig A in S1 File. Compartments and rates for TB transmission model. Fig B in S1 File. TB transmission model stratified by Body Mass Index. (DOCX) [file pone.0128187.s001.docx]

**File S1:**

**Model overview:** We created a dynamic deterministic compartmental model of the TB epidemic using methods similar to those previously described in the literature [[1](#_ENREF_1)]. Difference equations were used in order to take into account two simultaneous processes that change with time: the infectious process and the change in weight distribution in the population. The model classified the population by TB infection/disease status (Fig. A), and by BMI strata (Fig. B). In the main text we presented an overview of the model together with details of the parameters (Table 1a and 1b). More detail of the TB transmission model is provided below.

**TB transmission model:** The population was categorized according to their TB infection/disease status into the following states (Fig. A): susceptible (S) , latent- fast (ie. progression to disease from recently acquired infection) (L_f_) and latent (ie. reactivation from longstanding infection to disease) (L_s_), infectious (I), on treatment (Tx) and re-infected (L _re-inf_). Individuals enter the model as susceptible to TB infection at a rate calculated from the projected population growth rate for India between 2010 and 2030 and the birth rate [[2](#_ENREF_2)]. Individuals exit this compartment with TB infection or death. Of those who acquire TB infection, a small fraction progress to TB rapidly [[3](#_ENREF_3)] [[4](#_ENREF_4)] and the remainder transition to latent infection where they have a much lower risk of reactivation [[4](#_ENREF_4)]. Those with latent infection are at risk of re-infection. Some of those who are successfully re-infected progress rapidly to TB disease, however this happens at a reduced rate relative to the initial experience due to the protection conferred by the first infection [[3](#_ENREF_3)] [[5](#_ENREF_5)]. Individuals with active TB may be diagnosed [[6](#_ENREF_6)] [[7](#_ENREF_7)] [[8](#_ENREF_8)] and start effective chemotherapy. The majority of those who receive treatment cure [[9](#_ENREF_9)] [[6](#_ENREF_6),[7](#_ENREF_7)], but some remain infectious or may die while on treatment [[9](#_ENREF_9)] [[6](#_ENREF_6),[7](#_ENREF_7)]. Infectious individuals can also spontaneously resolve [[5](#_ENREF_5)]. Death from other causes can occur from each compartment [[2](#_ENREF_2)]. In India, TB diagnosis and treatment is available through either the public sector or private sector. Additional detail on diagnosis and treatment by sector is provided below (See section on Additional Detail on TB program in India).

Individuals were also classified by body mass index (see next section) but not by drug resistance or HIV co-infection, due to low levels of both of these epidemiologic modifiers in this setting [[10](#_ENREF_10)] [[11](#_ENREF_11)].

**Incorporating Body Mass Index:** In addition to TB infection/disease individuals were also classified according to body mass index (BMI), an important risk factor known to impact the risk of tuberculosis incidence [[12](#_ENREF_12)]. All compartments described above were stratified into four categories of BMI (Fig. B). The rates of reactivation and rapid progression to active TB increased by BMI strata. Rates obtained from the literature (as described above) were applied to the fourth (heaviest) quartile, and increased by BMI quartile (see table 4 in main text for more detail) using the association described in the literature [[13](#_ENREF_13)] [[12](#_ENREF_12)] and methods described in the main text. Mixing between BMI strata was random, as there is no literature to support other assumptions.

***Additional detail on Model Calibration:*** Observed TB incidence was used to calibrate the model to the setting of Central Eastern India. TB incidence for the time period and setting was obtained from self reported TB prevalence data from the North Eastern States as reported in the 2005 India Demographic Health Survey (DHS) [[14](#_ENREF_14)], together with estimated TB incidence trend data from WHO for India between 1990-2000 [[15](#_ENREF_15)]. The point estimate for TB prevalence in 2005 was 700 per 100,000, and the corresponding TB incidence at this time was calculated to be 350 per 100,000 (assuming 2 year survival). WHO data from pre- and post- 2005 was used to inform trends in TB incidence over the full 20 year period.

***Additional detail on transmission parameter:*** As described in the main text, we first fit a fixed transmission parameter calibrated to the equilibrium TB incidence reported by the WHO for the years 1990 to 2000 [[15](#_ENREF_15)]. We then allowed this parameter to decline linearly between the years 2001 and 2011 to correspond to the estimated decline in TB incidence during this period. The specific rate of decline for the beta was refined during the calibration process. This calibration procedure yielded a transmission parameter of 3.9X10^6^ in 2001 and 3.3X10^6^ in 2011. We assumed that incidence continued to decline at this rate until it was 2.2X10^6^ in 2030.

***Additional Detail on TB program in India:*** TB diagnosis and treatment in India is available in both the public and private sector. Further details on case detection and TB treatment by sector are provided below.

*Case Detection:* Case detection remained constant in India between 1990-2010 according to the World Health Organization (WHO) [[15](#_ENREF_15)]. WHO data was combined with an estimate from the literature of the case detection rate in the private sector of 0.2 per year [[8](#_ENREF_8)]. Estimates by sector were combined using data from Satyanarayana et al. on the proportion of cases treated in the private sector (52%) or in the public Revised National TB program (48%) [[7](#_ENREF_7)].

*TB treatment:* Treatment outcomes for the public sector data were obtained from the WHO Global Report between 2000 (82% treatment success) and 2010 (88% treatment success) [[15](#_ENREF_15)] . A linear trend was assumed in order to extrapolate estimates of treatment success over this period of time. For the private sector, treatment outcomes were assumed to remain constant over time and data was obtained from a study that considered TB treatment outcomes in private clinics in India, where treatment success was reported to be lower (72%) in 2002 [[9](#_ENREF_9)]. Failure/default rates were assumed to decline over time as cure rates increased. In both sectors, death while on TB treatment was assumed to remain constant. TB treatment outcomes by sector were again combined using data from Satyanarayana et al. on the proportion of cases treated in the private sector (52%) or in the public Revised National TB program (48%) [[7](#_ENREF_7)].

Fig A: Compartments and rates for TB transmission model


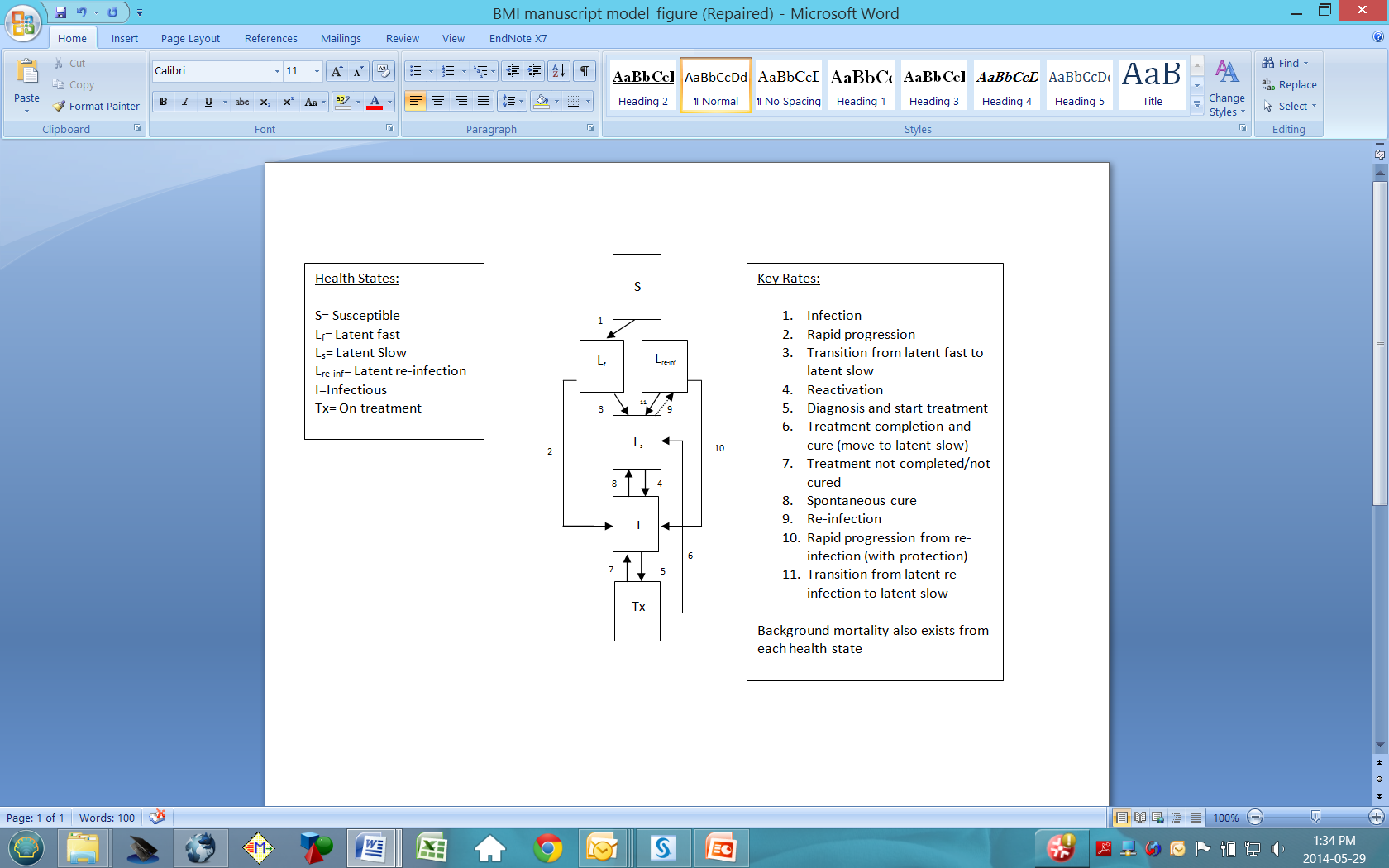


Fig B: TB transmission model stratified by Body Mass Index

p_x_= rapid progression rate , r_x_= reactivation rate (varies by BMI strata 1-4). All other rates as described in Fig A.


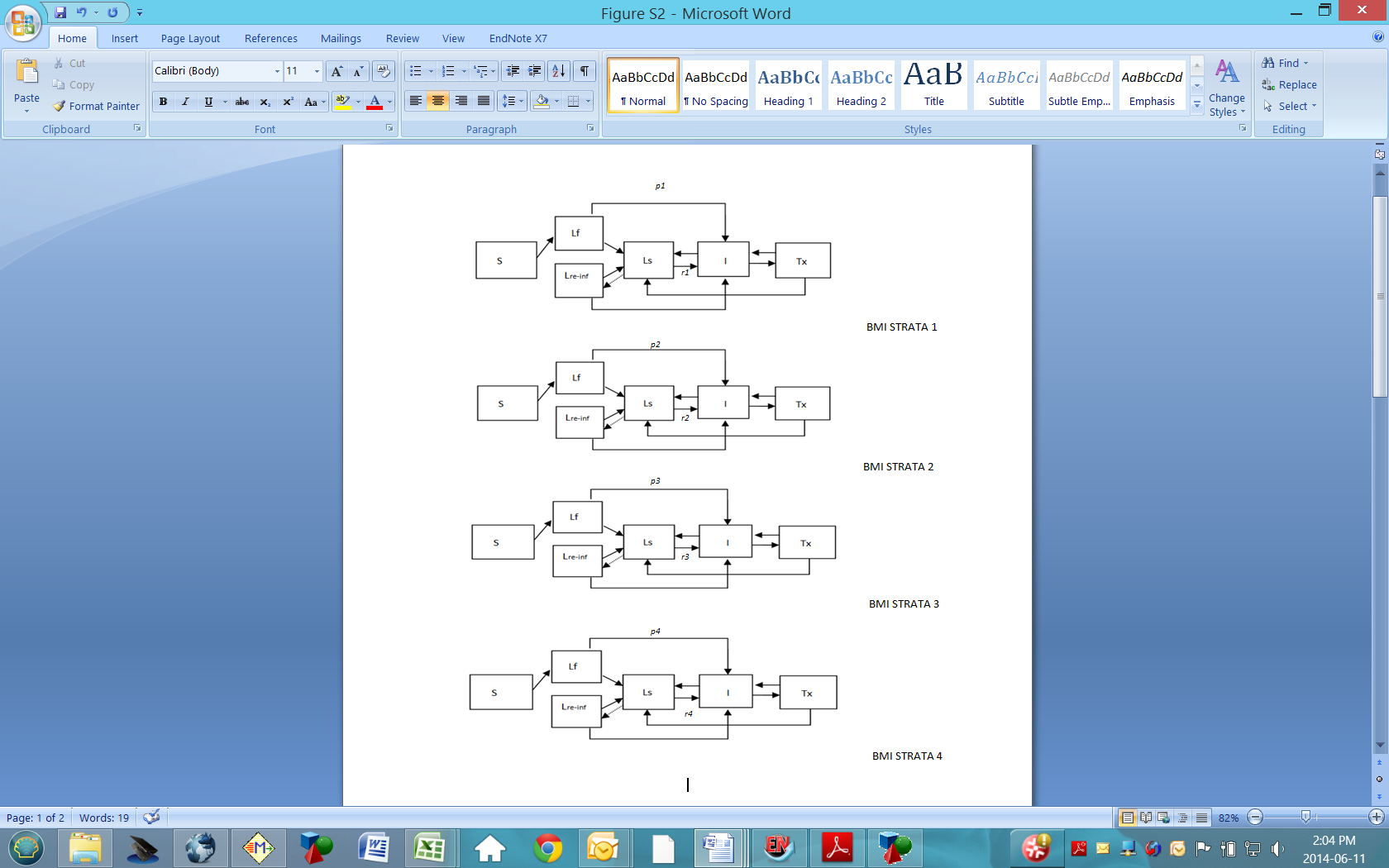


References:

1. Lin HH, Murray M, Cohen T, Colijn C, Ezzati M (2008) Effects of smoking and solid-fuel use on COPD, lung cancer, and tuberculosis in China: a time-based, multiple risk factor, modelling study. Lancet 372: 1473-1483.

2. United Nations Department of Economic and Social Affairs/Population Division (2013) World Population Prospects: The 2012 Revision, .

3. Vynnycky E, Fine PE (1997) The natural history of tuberculosis: the implications of age-dependent risks of disease and the role of reinfection. Epidemiol Infect 119: 183-201.

4. Dye C, Garnett GP, Sleeman K, Williams BG (1998) Prospects for worldwide tuberculosis control under the WHO DOTS strategy. Directly observed short-course therapy. Lancet 352: 1886-1891.

5. Sutherland I, Svandova E, Radhakrishna S (1976) Alternative models for the development of tuberculosis disease following infection with tubercle bacilli. Bull Int Union Tuberc 51: 171-179.

6. World Health Organization (2012) Global Tuberculosis Report 2012. Geneva.

7. Satyanarayana S, Nair SA, Chadha SS, Shivashankar R, Sharma G, et al. (2011) From where are tuberculosis patients accessing treatment in India? Results from a cross-sectional community based survey of 30 districts. PLoS One 6: e24160.

8. Menzies NA, Cohen T, Lin HH, Murray M, Salomon JA (2012) Population health impact and cost-effectiveness of tuberculosis diagnosis with Xpert MTB/RIF: a dynamic simulation and economic evaluation. PLoS Med 9: e1001347.

9. Arora VK, Sarin R, Lonnroth K (2003) Feasibility and effectiveness of a public-private mix project for improved TB control in Delhi, India. Int J Tuberc Lung Dis 7: 1131-1138.

10. Department of AIDS Control, National AIDS Control Organization, India. http://naco.gov.in/NACO/Quick_Links/HIV_Data/ India HIV Data. Access date July 10th 2014.

11. World Health Organization (2008) Anti-tuberculosis drug resistance in the world: the WHO/IUATLD Global Project on Anti-Tuberculosis Drug Resistance Surveillance. Fourth global report. Geneva.

12. Lonnroth K, Williams BG, Cegielski P, Dye C (2010) A consistent log-linear relationship between tuberculosis incidence and body mass index. Int J Epidemiol 39: 149-155.

13. Cegielski JP, Arab L, Cornoni-Huntley J (2012) Nutritional risk factors for tuberculosis among adults in the United States, 1971-1992. Am J Epidemiol 176: 409-422.

14. Demographic Health Survey. www.measuredhs.com. Access date July 10th 2014

15. World Health Organization (2013 ) Global Tuberculosis Report 2013. Geneva.
